# Model of Diagnosing and Searching for Incompatibilities in Aluminium Castings

**DOI:** 10.3390/ma14216497

**Published:** 2021-10-29

**Authors:** Andrzej Pacana, Karolina Czerwińska

**Affiliations:** Faculty of Mechanical Engineering and Aeronautics, Rzeszow University of Technology, Aleja Powstancow Warszawy 12, 35-959 Rzeszow, Poland; k.czerwinska@prz.edu.pl

**Keywords:** mechanical engineering, ultrasonic testing, eddy current testing, quality engineering, cracking mechanics, quality management

## Abstract

An essential element of any industry is castings, which is determined by the technical conditions for their reception. However, conducting production in the foundry technology is burdened with many difficulties associated with, for example, the inability to control all of the parameters that may affect the casting quality. Therefore, it is essential to undertake improvement actions in this area. Efforts are being made to use non-destructive testing (NDT) as a part of quality control, but these methods are rarely combined in a single diagnostic run. As a part of quality improvement, it is also essential to identify the root cause of the problem. For this reason, it is justified to develop a model of diagnosing and searching for non-conformities, which would combine NDT tests and quality management tools. The model included the visual, ultrasound, and eddy current examination in the diagnostic part, and the Pareto–Lorenz diagram correlated with ABC method, histogram, and 5WHY method (asking five questions why). The study’s originality is manifested in the combination of several NDT methods with quality management methods in one model. Using integrally configured methods in the proposed model, it was possible to: reduce diagnostic uncertainty, characterize the critical group of non-conformities, and identify the root causes of the quality problem. The model is a new and universal method that can be implied in any foundry company in order to ensure the stability of the production processes. The application of the model contributes to an increase in the detection speed and enables the reduction of non-conformities in aluminium castings, thus increasing the quality level of the offered products.

## 1. Introduction

One of the most critical objectives of running a company is to make as much of a profit as possible, which depends mainly on the number of orders. Today’s foundry companies are looking for ways to ensure their continued success in the marketplace while creating a sustainable livelihood and prestige [1,2,3,4]. The availability of goods and services in the market makes product quality a major factor in competitiveness. The competitiveness of any national economy is based on the competitiveness of its enterprises, which are elements that take into account its characteristics to have a grasp on the components of competing [5,6,7].

Essential for each industry are castings, the quality of which is determined by the technical conditions for their reception. The production of casting, and thus maintaining the high quality of the finished product, is associated with some technological parameters that can affect the quality of the finished product [8,9,10]. The problem occurring during the casting production process is the impossibility of simultaneous control of all factors of the technological process [11]. Running production in foundry technology is fraught with many difficulties. A wide variety of defects occurring in castings results from the very essence of the technology of castings production, consisting of technological operations including the design and manufacture of the casting mould and the technology of melting the liquid metal [12,13]. One of the essential components affecting the quality and competitiveness of the casting is to confirm that the casting is free of defects [14,15]. Any deviation of the features of the structure, material, and physicochemical or mechanical properties from the applicable requirements is considered a defect or flaw in castings [16,17]. When producing castings, it is impossible to ensure that there are no defects in every piece produced. Proper fabrication of aluminium castings is significant due to the vast possibilities of casting to produce various structural parts. The use of casting technologies allows for the manufacture of details with a complex structure that are components of vehicles, machinery, equipment, or measuring instruments [18,19,20,21,22,23]. Considering the progressive development in the complexity of structures and the associated increase in requirements for the components used, it is necessary to conduct material and technical research. In the context of ensuring the desired quality of workpieces, the vital issue is to control the execution of the process itself and the correctness of castings manufacturing [8,24]. Hence, it is reasonable and necessary to select not only technological parameters aimed at stabilizing the process, but also to select appropriate control methods (inter-operational control and final control).

In order to control the quality of castings, the authors of many scientific papers indicate the possibility of non-destructive testing (NDT), which allows for the identification of the possible inconsistencies without significant influence on its structural and surface properties [25,26,27,28,29]. Non-destructive testing is widely used in aviation [30], automotive [31,32,33], railway [34], oil and gas [35], pipe inspection [36,37], bridges [38,39], and the characterization of defects in ceramics [40], among others. These studies show how advances in technology have broadened the application of non-destructive testing even in industries that may not be manufacturing-oriented, noting that NDT is not limited to anomaly detection. Although advances have improved the way testing is performed and increased the benefit from its use, it is essential to remember that these techniques are primarily manual and rely heavily on the knowledge and experience of inspectors, which leaves room for error [41]. Therefore, more and more often, the authors of studies pay attention to determining the optimal design of castings in the context of facilitating the implementation of non-destructive testing tests with specific methods [42]. In addition, efforts continue to increase the speed of inspections, reduce the preparation required, and, where possible, carry out inspections without shutting down work [43]. Authors [44,45] in the framework of test automation consider the possibility of applying computer science principles in non-destructive testing according to the paradigm of industrial enterprises based on Industry 4.0. Considerations on the adaptation of NDT to the reality of Industry 4.0 are also carried out in many other studies, which indicate the opportunities, barriers, and chances of non-destructive testing adaptation [46,47,48,49,50]. The possibility of integrating external data acquisition instrumentation with industrial robots to improve the speed, accuracy, and repeatability of NDT inspection is also presented [51,52].

The analysis of publications related to the subject of aluminium castings inspection shows that attempts are being made to improve the performance of casting evaluation using single NDT methods. However, it should be remembered that if the scope of work is simple, it is permissible to use one testing method, but when we come to the control of castings with complex geometry and a great variety of shapes, often with a significant degree of complication, it happens that a single testing method does not provide sufficient information about the desired quality of the casting [53]. Thus, it is reasonable and necessary to combine different NDT methods, which is sparsely found in the literature to perform stationary (e.g., [54,55]) or field testing (e.g., [56]). The NDT methods combined in quality control should be complementary. An example is the implication of ultrasonic testing with eddy current detection. Although these methods have the advantages of no contract requirement, fast detection, and simple signal processing, there is a blind area near the surface in electromagnetic ultrasonic detection, and pulsed eddy current detection cannot accurately detect deep non-conformity locations. However, these two techniques can potentially be integrated with the control process to overcome their limitations [57,58]. In addition, both eddy current and ultrasonic testing are often indicated by the literature as effective in identifying cracks in aluminium castings, which are often encountered by foundries [59] or in determining the thickness of coatings [60].

In the literature, there are studies indicating the use of non-destructive testing including eddy currents and ultrasonic testing for non-cast products. The eddy current method is also used, for example, in the inspection of pipes (diameter [61] and thickness measurements [62]), welding zones [63], and thickness measurements of composite materials [64]. Diagnostics with the use of the ultrasound method is also characterised by wide application possibilities. This method is used, for example, to inspect concrete elements [65] and their joints [66], to characterize rock material [67], and to inspect welded joints [68].

Performing an adequate identification of non-conformities in aluminium casting is one of the steps in the context of improving the casting process. To ensure process stability, the causes of identified non-conformities should be eliminated through analysis and corrective actions [69,70,71]. Authors [72,73,74,75] point out the benefits of combining a range of hardware and software resources of NDT technology to transfer data to the cloud or network, where the data is analysed, stored, processed, evaluated, predicted, and fed back, providing a convenient foundation to undertake quality analysis and stabilize the casting process. Hence, it is reasonable and necessary to select technological parameters that improve the process and select appropriate quality management instruments contributing to the achievement of the desired quality level and organisational and financial benefits [76,77,78,79,80]. The Pareto–Lorenz diagram and the ABC method (ABC classification) are used to identify the most critical group of factors that generate a given problem [81,82]. After specifying the most important factors, it is advisable to present the structure of critical sets of variables (empirical distribution of characteristics) using a histogram, which will facilitate the analysis process by visualising the frequency of occurrence of numerical data [83]. As a part of further analysis of the causes of quality problems, it is reasonable to use the 5WHY method (asking five questions why), which allows you to get to the source of the disturbance, thoroughly investigate its cause, and focus on solving it effectively [84].

The literature on quality management often presents the issue of quality analyses supported by single quality management tools [85,86,87,88,89,90], or develops models in production process optimization [91,92]. However, no integrally configured quality control method has contributed to the realization of in-depth causal analysis of manufacturing non-conformities identifying the root cause of non-conformities. However, the authors did not find literature studies presenting integrally configured methods of quality control, which would contribute to the implementation of an in-depth causal analysis of manufacturing non-conformities identifying the root cause of non-conformities.

Taking into account the indications from the literature, it can be concluded that there is a research gap in terms of combining non-destructive testing with quality management techniques in the framework of the defect analysis of aluminium castings. There is also a lack of a model for diagnosing and looking for inconsistencies in aluminium castings. Therefore, it is reasonable to develop a sequence of non-destructive testing use, with quality management tools in which the output from one tool is the input to the next.

The research aimed to develop a model of an integrally configured method for diagnosing and searching for non-conformities in aluminium castings consisting of the integration of non-destructive tests visual method, eddy current, and ultrasound method, and quality management techniques occurring in succession (Pareto–Lorenz diagram correlated with the ABC method, histogram, and 5WHY method) to be used in post-casting process control or inter-operational condition quality control of the surface of castings. An additional objective, as part of the improvement activities, was also to perform a test of the proposed model in one of the foundry companies. 

## 2. Model

**Figure 1:**
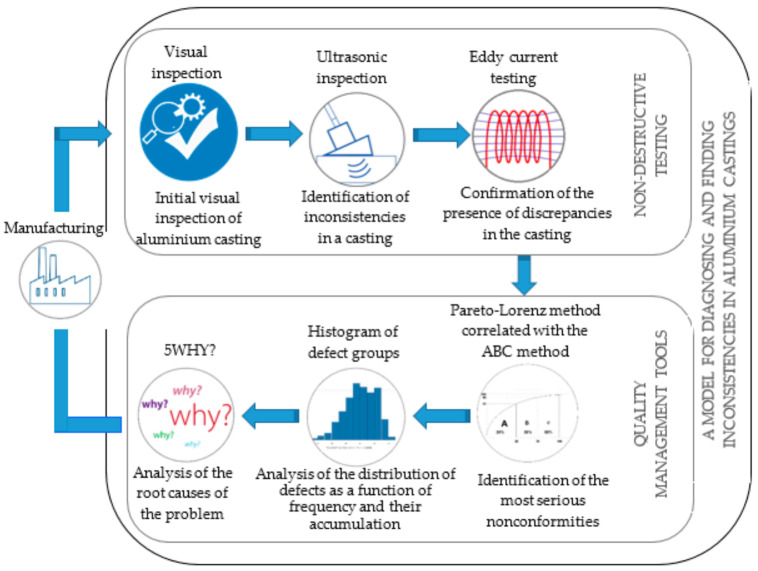
General scheme of the model of diagnosing and searching for non-conformities in aluminium castings indicating the justification of the choice of methods in the context of continuous quality improvement.

Numerous problems concerning the assurance of a good quality of cast products force us to search for different diagnostic methods. As a part of identifying non-conformities arising after the casting process, it is possible to apply non-destructive testing (NDT). These are testing techniques used to detect non-conformities and determine the condition of the material in a non-destructive manner for the product being inspected. However, the result of a single test with the application of the NDT technique does not ensure the absence of defects in the product; therefore, it is recommended to apply more diagnostic methods located one after another within the framework of quality control. In the context of the implementation of improvement actions (quality analysis) allowing for the analysis and elimination of identified non-conformities, a combination of quality management tools may be used, where the output from one tool will constitute the input to the subsequent, more in-depth analyses. A general schematic of the model for diagnosing and finding non-conformities in aluminium castings indicating the rationale for the choice of methods is shown in Figure 1.

The model was developed as a stage control (diagnostic-analytical) implemented after a unique process of aluminium casting, allowing it to go beyond the passive control. The model was divided into two areas: the first relating to the detection of non-conformities in aluminium castings, and the second relating to the analysis of the causes of the problem.

Methods from the non-destructive testing group and quality management tools were used in the model because they are preferred in casting quality control and quality problem analysis. Many cited literature items confirm the effectiveness of applying the considered methods individually [41,85,86,87,88,89,90,93,94,95,96,97].

Non-destructive testing is a group of testing methods that provide information about casting properties without significantly affecting their structural and surface properties. The overriding aim of non-destructive testing is primarily to detect inconsistencies that have the character of discontinuities of material. The use of non-destructive testing in the proposed model is also justified by safety considerations and the economic aspect of an unforeseen failure [25,26,27,28,29,41].

The literature on the subject indicates that visual testing, as a rule, should be carried out as the first in the sequence of tests performed—a preliminary test [98,99,100] to which attention was paid during the development of the model. The visual method is one of the most important inspection methods despite the developed information technologies and very high automation of the production process itself. Visual inspections of the product are still used to ensure quality with insufficient process stability, and despite the increased use of image processing systems, there is no indication that the human factor will be eliminated from these areas of the company shortly [101,102]. The main objective of visual quality control is to separate defects visible to the unaided eye. An additional rationale for including visual inspection in the model was its benefits. Today, it is an economically viable measure that does not require expensive equipment and can be used practically in any area of the company [103,104]. 

The ultrasonic method (UT) is based on the phenomenon of propagation of ultrasonic waves in solids. In the tested sample, the propagating waves give a permeable signal (pass/shadow technique) or a signal reflected from the surface of discontinuity (echo technique), which returns to the head and, after being processed into electric vibrations, is observed as impulses on the oscilloscope screen or registered in the memory of the computer, coupled with the defect score [105,106]. Ultrasonic testing was added to the model of diagnosing and searching for discrepancies because of the possibility of detecting dangerous flat and narrow-slit discontinuities with its use, which were difficult to recognize by, for example, X-ray method. In addition, this method makes it possible to precisely locate both internal and external inconsistencies in a rapid manner (rapid implementation of tests and rapid acquisition of results) [107]. Another fact supporting the use of the UT method was that it creates the possibility to perform diagnostics with one-sided access to the tested object, which is extremely important considering the specificity and constructional quantity of complicated details produced in the foundry industry [108].

Eddy current testing involves inducing an alternating electromagnetic field in the material under test and receiving the material’s response to such actions [109]. The literature on the subject indicates that this method, one of the NDT techniques of the highest reliability and very high sensitivity, is used in the production control systems, among others in the foundry industry, where the dermatoscopic diagnosis of materials is particularly responsible where parts of the complicated shape are realised. Authors of papers [110,111,112,113] point out the significant number of advantages of the eddy current method, which include: high speed of testing and interpretation of results, the possibility of testing all metals, and additional non-contact testing (no need for a coupling agent between the probe and the tested surface). The ability to perform tests without removing the protective coatings of the product and the ability to perform the test through metallic coatings other than the native material of the product are also definite advantages [114]. The presented advantages confirm the validity of using eddy current testing as a second detection test in the model.

Quality management in organisations requires control of the foundry processes to identify the defects occurring and in-depth analyses concerning the source causes of the inconsistencies, which is possible thanks to quality management tools [22,69]. An important issue is identifying problems by prioritising them according to the most important causes, which is enabled by the Pareto–Lorenz diagram correlated with the ABC method. Thanks to the correlation of both tools, it is possible to quickly identify priorities and skilfully manage resources in order to eliminate one group of key causes, which allows for an avoidance of the dispersion of resources to all causes at one time, which reduces the effectiveness of activities [81,82]. When conducting qualitative analyses of variables from the group of crucial causes, it seems helpful to obtain information on the empirical distribution of the trait. This procedure, i.e., presenting the obtained results for certain variables, can be illustrated using a histogram. A clear illustration (in a graphical way) of the values at which most of the results are located can be pointed out as an advantage of such a procedure [115]. Translating to the ground of casting discontinuities research, it seems reasonable to identify the quantities within the limits of which the most significant number of discrepancies occurs, thus defining the structure of analysed variables. This allows further improvement activities to be tailored to the specifics of the most severe variables.

In order to identify the root cause of the non-conformity, the literature recommends using the 5WHY method [116]. Asking a few “why-is-that?” questions creates the opportunity to identify the source of the disturbance, thoroughly investigate its cause, and better understand the situation. The advantage of such an action is simplicity. The method does not require special preparation from the employees; however, it encourages analytical thinking and problem identification [117].

**Figure 2:**
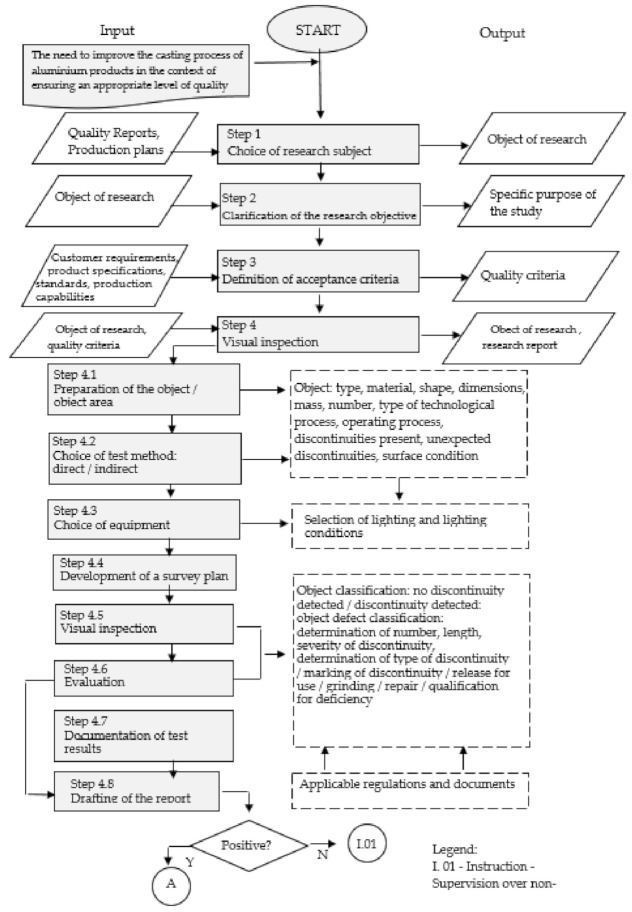
A model for diagnosing and finding non-conformities in aluminium castings—part 1. Own elaboration.

**Figure 3:**
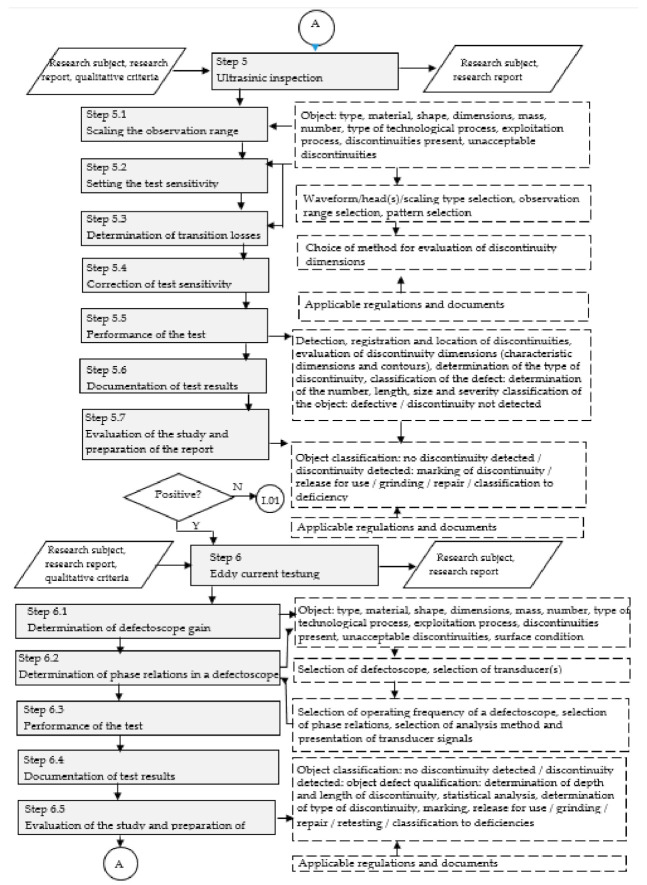
A model for diagnosing and finding non-conformities in aluminium castings—part 2. Own elaboration.

**Figure 4:**
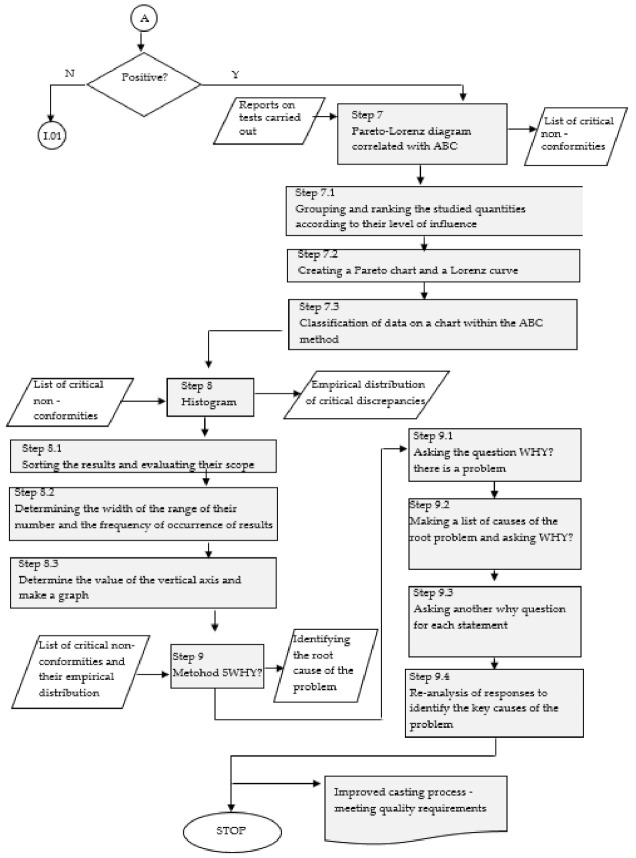
A model for diagnosing and searching for non-conformities in aluminium castings—part 3. Own elaboration.

A complete model for diagnosing and searching for non-conformities in aluminium castings was developed in nine main steps (Figure 2, Figure 3 and Figure 4).

In the following section, the characteristics of the model will be presented.

Step 1. Choice of research subject.

Due to the possibility of applying selected non-destructive testing in the model, the test object should be any axisymmetrical aluminium casting (or made of conductive material), which should be diagnosed in the presence of the most dangerous discontinuities: flat, narrow-slit, and other object discontinuities. 

Step 2. Clarification of the research objective.

The aim should be to improve the process of diagnosing and searching for non-conformities in aluminium castings according to the Kaizen concept, i.e., maintaining and improving the prevailing quality standards in foundry companies. 

Step 3. Definition of acceptance criteria.

The acceptance criteria should relate to the product to be selected in the first stage of the model. Quality criteria that characterize the product in terms of its user characteristics should be defined. The selected criteria should be correlated with customers’ requirements, production possibilities, and control possibilities. Additionally, they should be following the guidelines from standards and legal regulations.

Step 4. Visual examination.

This step is to perform a preliminary visual inspection. Visual testing within the model is carried out as a preliminary test before the ultrasonic testing of castings to detect non-acceptable defects for their repair before starting other expensive non-destructive tests. Visual examination is mainly carried out to detect the most dangerous discontinuities, which are surface discontinuities [100,101]. It is possible to detect cracks with a depth from about 0.1 mm in width to about 0.01 mm and a length of about 0.1 mm [104].

In ultrasonic and eddy current testing, the use of visual testing is required to observe the signals and images of identified discontinuities.

Step 4.1. Preparation of site/area of the site.

Within the framework of this stage, it is necessary to become thoroughly familiar with the object, including information concerning: the type of object, the material from which it was manufactured, its shape, geometry, mass, the number of pieces manufactured, the types of technological processes after which the test is carried out, and the process of exploitation of the product, as well as the type of identifiable discontinuities, including the condition of the surface before the test [98]. 

Step 4.2. Choice of test method: direct, indirect.

When choosing the type of method, attention should be paid to the availability of the surface to be inspected. Direct examination allows the diagnosis of surfaces that are directly accessible for visual inspection. This type of examination is performed with the unaided eye or with the use of magnifying glasses and microscopes [99]. However, post-medical examinations or optical examinations are performed using endoscopes, videoscopes, periscopes, and mirror sets [103].

Step 4.3. Equipment selection.

This stage refers mainly to the appropriate choice of illuminant and lighting conditions. The lighting requirements are based on the fact that it is unknown what discontinuities will be found before testing begins, so it must be assumed that these may be narrow-slit discontinuities [103].

Step 4.4. Develop a study plan.

The study plan should plan the study session. It is advisable to prepare, for comparative purposes, atlases of discontinuities of objects of given classes [99].

Step 4.5. Inspection, Step 4.6. Evaluation.

The indicated steps refer to the object classification, which informs the detection or not of a discontinuity in the tested object. Detected discontinuities are classified by their number, length, severity, and type. Subsequently, identified discontinuities shall be marked. On the basis of the collected information, the product may be: released for use, subjected to grinding, repaired, or classified as non-compliant and directed to deficiencies. 

Step 4.7. Documentation of test results, Step 4.8. Test report.

Documentation indicating unit test results shall be made during the visual inspection. However, for each batch of products inspected, a test report should be drawn up following the guidelines in force in the given company, including essential information describing the identified non-compliances. 

Step 5. Ultrasonic tests.

This type of model testing was used to diagnose internal, surface, and subsurface discontinuities in aluminium castings.

Step 5.1. Scaling the observation range, Step 5.2. Setting the test sensitivity.

The value of the observation range is influenced by the angle of the introduction of the ultrasound wave into the material. The observation range shall be determined so that the maximum route covering the entire test area is a maximum of 80 % of the observation range. When conducting tests with normal heads, it is recommended that the first two echoes of the bottom are visible on the screen and the second echo constitutes about 80% of the observation range [105].

Test sensitivity is the ability to detect specific discontinuities in the material from specific distances. Control standards (or reference samples) are used in determining the sensitivity of the test. The registration limits of discontinuities should be adopted following appropriate criteria, standards, technical conditions, or other binding requirements [106].

Note that, like the scaling of the observation range, setting the sensitivity of the test should be done before each test. Several parameters are relevant at this stage [108,118]:

- Wavelength, which describes the relationship:

(1) $$\lambda =\frac{c}{f}$$

where *λ* represents the wavelength (mm or m), *c* represents the speed of a given type of ultrasound wave in a given material (m/s or mm/µs), and ƒ represents the frequency of ultrasound transducer (Hz or MHz).

- Speed of wave propagation in the tested casting:

(2) $$c_{L}=\sqrt{\frac{E\left(1-v\right)}{\rho \left(1+v\right)\left(1-2v\right)}}$$

where *c_L_* represents the velocity of propagation of longitudinal waves [m/s], *E* represents the modulus of elasticity (Pa), *v* represents the Poisson’s number, and *ρ* represents the density of material (kg/m^3^).

- Transverse wave propagation velocity:

(3) $$c_{T}=\sqrt{\frac{G}{\rho }}=\sqrt{\frac{E}{2\rho \;\left(1+v\right)}}$$

where *G* represents the modulus of elasticity (Pa).

- Propagation velocity of surface waves:

(4) $$c_{R}=\alpha \sqrt{\frac{G}{\rho }}=\alpha c_{T}$$

where *α* represents the coefficient, and where:

(5) $$\alpha =\frac{0.85+1.12v}{1+v}$$

Step 5.3. Determination of transition losses.

Wave scattering processes cause scattering losses (reflection and refraction) at grain boundaries of product materials and fine inclusions, pores, and separations. Some of the energy of the wave beam is scattered, and propagates in different directions. An immense contribution to the attenuation of ultrasound waves is usually due to the loss of beam divergence. The attenuation coefficient α_1_ of ultrasound waves depends strongly on the frequency, as indicated by the Formula (6) [118]:

(6) $$\alpha_{1}=af^{2}+bf^{4}$$

where *α*_1_ represents the wave attenuation coefficient (dB/m), *a, b* represent the coefficients, and *f* represents the transmitter frequency (MHz).

The dependence (6) applies to waves with a length greater than three times the grain size [107]—thus, the dependence applies in the defectoscopy of the analysed left *l*. 

Dependence (7) recognises losses due to beam dissection and losses related to wave damping in the material [107]:

(7) $$20lg\left(\frac{p\left(z\right)}{p_{0}}\right)\;=const-20lgz-2\alpha_{t}z$$

where *p*(*z*) represents the pressure in the ultrasonic beam at distance “*z*” from the transducer (Pa), *p*_0_ represents the pressure generated by the transducer (Pa), *z* represents the distance from the transducer face along the acoustic beam axis (m), and *α*_1_ represents the ultrasonic attenuation coefficient (dB/m).

The definition of the attenuation coefficient *α1* of ultrasonic waves is given by the relation (8):

(8) $$\alpha_{1}=\frac{∆p}{l}$$

where ∆*p* represents the decrease of acoustic pressure on path *l*, caused by crowding of waves, and *l* represents the distance travelled by waves. 

The damping factor *α*_1_ faj is a characteristic quantity for the material, the frequency of the waves, and the type of ultrasonic waves. 

Step 5.4. Sensitivity correction.

Sensitivity correction is implemented due to transition losses. Accurate alignment of the camera and transducer is crucial as it affects the accuracy of imaging the ultrasound waveform in the tested object, and allows the accuracy of the discontinuity’s depth [107].

Step 5.5. Test execution.

The travel areas shall be determined before each test, considering the test technique, beam introduction angle, and weld and base material over a width of at least 10 mm. During the test, the probe shall be moved in such a way as to ensure the effectiveness of detecting discontinuities and to obtain maximum information for determining their type and size. The displacement area shall first be examined with a standard or angular probe in order to detect any defects that would make examination difficult or impossible [106,107,118].

The implementation of the ultrasonic test method includes such activities as:

- detection of discontinuities;
- registration of results;
- determining the location of discontinuities;
- assessment of the dimensions of discontinuities (evaluation of the length and height of the discontinuities and estimation of the width of the discontinuities); and
- determining the type of discontinuity.

Step 5.6. Documentation of test results, Step 5.7. Test report.

Development of the research documentation and the research report is performed analogically to the completion of Stage 4.7. and Stage 4.8.

Step 6. Eddy current testing.

Eddy current fetoscopy was used to inspect the castings for the presence of flat surface discontinuities, narrow-slit discontinuities, and relatively large near-surface discontinuities. It is possible to detect cracks with a depth of about 0.1 mm, a width of about 0.0005 mm, and a length of about 0.4 mm [110,112]. 

Step 6.1. Determine defect score gain, Step 6.2. Determine defect score phase relationships.

The selection of the operating frequency of the defect score provides the possibility to regulate the penetration depth of eddy currents, i.e., to select the areas to be inspected. High-frequency transducers (of the order of several MHz) are used for surface layer testing. On the other hand, lower frequency transducers (of the order of several to several hundred kHz) should be used to detect discrepancies and structural changes at a certain depth from the surface [111].

In connection with the fact that the aim of the control realised in the study is the detection of surface discontinuities of the casting, it is necessary to select possibly high frequencies of transducer operation. 

Step 6.3. Test execution.

The phenomenon of eddy current relation was used in resectoscope. Eddy currents induce a magnetic field that overlaps the magnetic field induced by the coil. Maxwell’s first and second equations can describe this phenomenon [119]:

(9) $$rot\;H=\gamma E+\varepsilon \frac{\partial E}{\partial t}\;\;\;\;\;\;$$

(10) $$rot\;E=-\mu \frac{\partial H}{\partial t}\;\;$$

where *E* represents the electric field strength, *t* represents the time, *H* represents the magnetic field strength, *ε* represents the electric permeability, and *µ* represents the magnetic permeability.

For harmonic excitation, Equations (9) and (10) take the following form:

(11) rot H=γE+jωεE    

(12) rot E=−jωμH  

If the inequality is satisfied:

(13) rot H ≫jωε  

Equation (11) simplifies to:

(14) rot H=γ E  

Then, the alternating magnetic field induces conduction currents, many times larger than the offset currents. Thus, conduction currents are included and offset currents are ignored. When the inequality is satisfied:

(15) γ≪ ωε

Equation (11) is simplified to the form:

(16) rot H=jωμE  

In this case, only the displacement currents induced in the material are considered. Regardless of the phenomenon we are dealing with (whether both types of currents (11) or only one of them (13) or (15) must be taken into account), the principle of defectoscopy is the same. The distribution of the resultant magnetic field depends on the homogeneity or non-homogeneity of the material under test. The inhomogeneities in the material under test cause non-uniform eddy currents, and induce a non-uniform magnetic field of the reaction [119].

Step 6.4. Documentation of study results, Step 6.5. Evaluation of the study and preparation of the report.

The protocol (report) from eddy current defectoscopy should include: a description of the product, a description of the inspection objective, an object of testing, acceptance requirements, a description of the testing method and technique, a description of the apparatus and settings of the apparatus, a description of the standards used for testing, evaluation of the test results (including drawings of the transducer signal trajectories, if any, for the detected discontinuities), the length of discontinuities, and, possibly, characteristics of discontinuities and an evaluation of the object quality following the requirements [111].

The eddy current tests given in the step are important, but the result of the tests is decided by the person who performs the tests and evaluates the product. 

Step 7. Pareto–Lorenz diagram correlated with ABC method.

The Pareto–Lorenz Diagram correlated with the ABC method was used in the model in order to identify the critical group of non-conformities (non-conformities with the highest frequency of occurrence and the most serious consequences).

Step 7.1. Group and rank the test quantities according to their level of influence, Step 7.2. Create a Pareto chart and Lorenz curve.

In order to make a Pareto–Lorenz diagram, we need to collect a sufficient amount of data about the phenomenon under study. Then, it will be possible to distinguish several categories of causes generating the analysed problem.

Rank the causes of the phenomenon under consideration from the most to the least significant, based on their frequency of occurrence. In this way, it is possible to prioritise and set a direction for action. By determining the cumulative values of each cause, we obtain ready data for drawing a bar chart with a Lorenz line.

Step 7.3. Classify data on a chart using the ABC method.

This step evaluates the values of the variables under study by their contribution to the value of their total annual consumption. In group A (critical inadequacies), variables are reaching the value of 75–80%, group B within 15–20%, and group C with a value of about 5%. The variables classified in this group have the largest share in the number of variables (60–80%) and a meagre share in the value (about 5%) [81,82].

Step 8. Histogram.

A histogram showing the distribution of defects as a function of the frequency of occurrence and its accumulation within the model should be made. The information contained in the histogram is helpful to characterize non-conformities and determine their type [115].

Step 8.1. Sort the results and evaluate their range.

Count the number of results and the number of occurrences of the trait, and determine the validation of a trait.

Step 8.2. Determine the number range width and frequency of results.

This step consists of determining the number of compartments, where the number of classes depends on the size of the analysed community and is determined as [115]:

(17) $$k=\sqrt{n}$$

where *k* represents the number of classes, and *n* represents the size of the tested population.

The range, on the other hand, is determined by the difference between the highest and lowest values of the characteristic (the so-called range), divided by the number of classes, which is calculated as follows [115]:

(18) $$c=\frac{\left(x_{max}-x_{min}\right)}{k}$$

where *c* represents the width of intervals, *x_max_* represents the highest value of a feature, *x_min_* represents the lowest value of a feature, and *k* represents the number of classes.

Step 8.3. Determine the value of the vertical axis and plot it.

According to the results obtained (the width of the range of numbers and the frequency of the occurrence of results), a graph should be drawn with the clarity of the data presented. 

Step 9. The 5WHY method (asking five questions why).

The purpose of the 5WHY method is to determine the true cause of the defect beyond a simple symptom diagnosis [116]. 

Step 9.1. Ask why there is a problem, Step 9.2. List the root causes of the problem and ask why, Step 9.3. Ask another why question for each statement, Step 9.4. Review the responses to identify the root causes of the problem.

The path used to locate the true causes of the problem in the 5WHY method is iterative (repeated five times or as long as necessary: five is just a conventional number). Continue the iteration until the cause is identified or there is a loop in the responses [117,118].

The developed model for diagnosing and searching for non-conformities in aluminium castings takes advantage of the diversity, complementarity, and redundancy of different methods and techniques in order to develop more reliable synergistic systems, increase the speed of detection, and enable the reduction of non-conformities in aluminium castings, thus increasing the quality level of the offered products.

The model helps to reduce the level of diagnostic uncertainty. Due to the varied quality and availability of data and the diverse nature of the sources, the results of the analyses are inherently uncertain. Relatively simple mathematical concepts (included in quality management tools) are adequate to describe the problem under consideration. Statistical approaches allow for a more rigorous assessment of model uncertainty and sensitivity [119,120]. The staged treatment of the analyses performed is intended to reduce uncertainty in both quantitative and qualitative terms [121]. The methodical action and stepwise framework proposed in the model allow for a systematic way to take into account the information collected and to obtain results as precise as the data suggest.

## 3. Assumptions and Limitations of the Model

The assumptions of the research model refer mainly to the possibilities that result from the applied non-destructive methods and quality management methods. The following assumptions were identified in the model as contributing to the effectiveness of the method:

- Prepare, for comparative purposes, atlases of discontinuities of the studied objects.
- Ensure adequate lighting (light: white, natural, artificial).
- Provide the ability to change the direction of illumination over a wide range.
- Running diagnostics is not dangerous for operators.
- You are possibly using the model at any stage of the production process (preliminary, inter-operational, final control).
- Applicability of the model to products made of electrically conductive materials (ferromagnetic and non-ferromagnetic metal and some composite materials).
- It is possible to perform non-contact or contact eddy current defectoscopy.
- Provision of a person experienced in the interpretation of NDT test results.
- Provision of reference systems (specific benchmarks).
- Availability of data on study results.
- Availability of acceptance criteria for analysed aluminium castings.

Implying the proposed model for diagnosing and searching for non-conformities in aluminium castings, one should consider its following limitations:

- Difficulty in testing products with rough surfaces, non-uniform products, and irregular shapes.
- Boundaries in the study of microscopic and thin objects. The ultrasonic examination enables the precise location of the defect if the indications are not complicated by the shape of the examined object, causing shape echoes to appear. Small wall thicknesses cause the phenomenon of internal wave reflection.
- In the case of ultrasonic tests, using a coupling medium is necessary to ensure the transfer of energy from the transducer to the boekt in tests using piezoelectric transducers.
- No detection of planar discontinuities along the ultrasound beam axis.
- Difficult examination of high-temperature objects due to melting of plastic heads and temperature dependence of the ultrasound wave propagation speed in the material.
- Difficulty of eddy current testing of ferromagnetic components due to the so-called skin effect (concentration of eddy current field near the surface).
- Limiting the depth of verification of material under eddy current inspection.
- Difficulty in detecting discontinuities (e.g., material delamination, cracks) lying parallel to the course of the probe winding and the test direction.
- Variable penetration depth during the implementation of eddy current testing.
- Vibration and impact make it difficult to find defects.

The indicated assumptions and calculations have been taken into account during the process of verifying the model of diagnosing and finding incompatibilities of aluminium castings.

## 4. Model Verification and Results

The study concerned batches of products made in the second and third quarter of 2020 in one of the manufacturing companies in Poland’s southern part. The scope of the inspection of the aluminium casting included verification of the casting surface, determination of the place of non-conformity occurrence, and precise determination of the type of identified non-conformity. Quality control also included checking the correctness of the casting marking. Quality control was performed following the steps indicated in the developed model of diagnosing and searching for non-conformities, respectively, to each production order.

**Figure 5:**
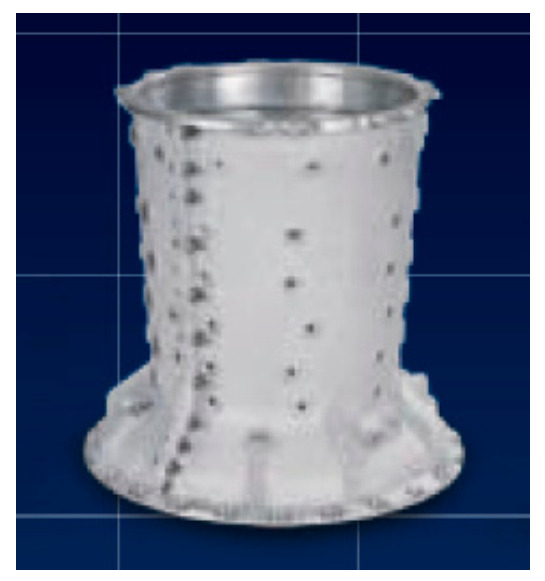
Subject of research—3D model of the compressor body.

An experimental study was conducted to evaluate the ability to detect internal inconsistencies in the product material. The object of research was a body casting of a compressor used in the mining industry. A 3D model of the compressor body is shown in Figure 5.

The compressor body is cast in AlCu4Ti alloy. The finished product has the following dimensions: 319 × ø339, and weighs 4.75 kg.

AlCu4Ti alloy is characterised by excellent machinability, good polishing properties, good weldability, and limited corrosion resistance (Table 1). The mechanical values of the alloy can be greatly varied by modification of artificial ageing. Due to the mentioned characteristics, the alloy is used for highly loaded parts where corrosion properties are not obstacles. This alloy has found applications in the automotive, engine manufacturing, me-too engineering, textile, defence engineering, and mining industries [122].

Properties for real castings, cooled from an elevated temperature shaping process and naturally aged.

Chemical composition of AlCu4Ti alloy is shown in Table 2.

Visual inspections in the company are performed by employees after each technological operation.

**Figure 6:**
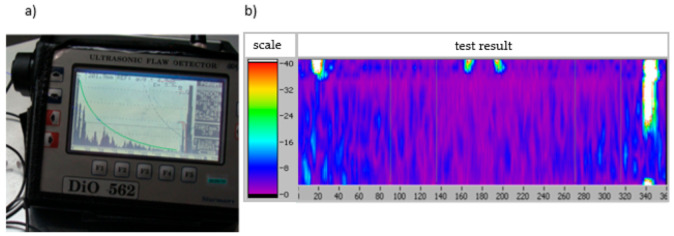
Example of the results of: (**a**) ultrasonic inspection and (**b**) eddy current inspection, indicating the presence of cracks in an aluminium casting.

Testing was carried out using the ultrasonic method and the eddy current method as a part of cyclic quality control tests during product manufacturing. The defectoscopic indications of both non-destructive methods are shown in Figure 6.

In this study, samples for metallographic examination were cut from the defective areas of the casting on a metallographic cutter and then embedded in resin. The next step was to sand and polish the samples with Saphir 530. Metallographic samples were etched with a 5% aqueous solution of HF acid. The microstructure was observed on a Zeiss Neophot 2 metallographic microscope.

**Figure 7:**
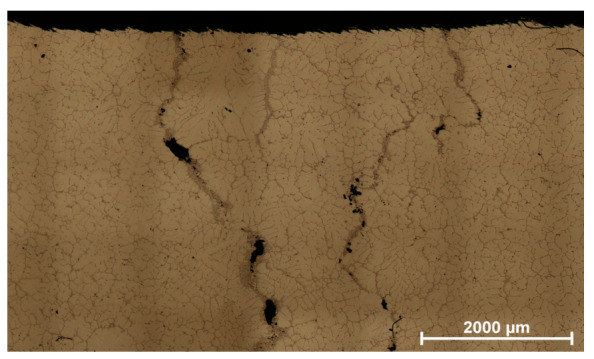
Microstructure of non-conformity area in a compressor body casting crack along segregation in AlCu4Ti alloy.

An example of the most frequently identified non-conformity cracks along the segregation in AlCu4Ti alloy is shown in Figure 7.

The discontinuity presented is unacceptable and thus qualifies the product for disposal.

**Figure 8:**
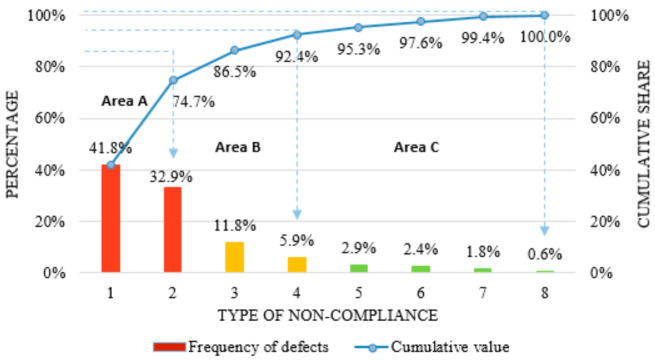
Pareto–Lorenz diagram supplemented by the ABC method for the non-conformities present in the tested batch of castings.

Defectoscopy with the application of NDT methods used to examine the analysed batch of products enabled the development of a Pareto–Lorenz diagram supplemented by the ABC method to determine the most fundamental frequency of occurrence from this point of view and the effects of the presence of certain non-conformities. The presentation of results through the indicated tool enables a practical solution of the quality problem thanks to the hierarchy of causes and priorities, allowing for the minimization or elimination of non-conformities. The result of the statistical analysis is shown in Figure 8.

On the Pareto–Lorenz diagram, the inconsistencies are marked in the following order: 1—cracks; 2—the presence of rows; 3—contraction cavity; 4—inclusions of a foreign material; 5—exfoliation; 6—presence of oxides (void); 7—crowing; 8—dimensional discrepancy; 9—under delivery; and 10—shape mismatch.

**Figure 9:**
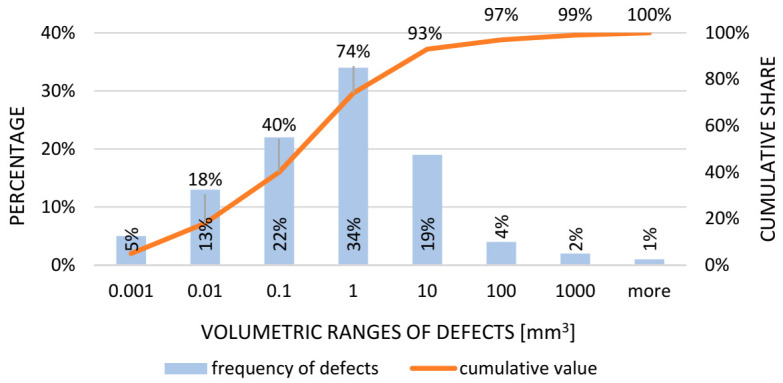
Histogram showing the frequency of occurrence of specified groups of defects (cracks).

In Figure 9, area A indicates the critical non-conformities (most frequent and having the worst effect), area B indicates the non-conformities with a medium effect, while area C includes non-conformities with negligible effect on the quality level of aluminium castings. The horizontal axis indicates the types of non-compliance analysed. On the other hand, the vertical axis on the left indicates the percentage inclusion of the studied inconsistencies (columnar layout). The vertical right axis indicates the cumulative percentage of the study variables (Lorenz line).

The results presented in Figure 9 will be analysed in two stages because, within the critical group of non-conformities, two types of defects were identified: cracks and ruptures in the aluminium casting.

Based on the Pareto–Lorenz diagram, we can see that cracks are the most frequently identified inconsistencies. The most stressed area where crack initiation and propagation most frequently occur is the base circle of the compressor body. This is due to the design of the casting (the presence of a large number of fixing holes) and the specific operating conditions of the product. The development of fatigue cracks in the analysed castings usually started from one side of the base flange—in the area of the highest stress concentration. The second major cause of loss is the presence of lint in castings. This inconsistency is most often found in the body mount area. The application of the Pareto–Lorenz diagram correlated with the ABC method made it possible to identify 25.3% of the non-conformities (cracks, presence of rutting), which cause 74.7% of losses. 

As part of an in-depth analysis of the size of discontinuities located inside the tested castings, a histogram was developed, showing the distribution of defects as a function of frequency of occurrence and its accumulation (Figure 9).

As shown in Figure 8, the most significant number of internal non-conformities generated during compressor body casting occurs in the size of about one mm3 and combined with defects in the range of 0.001 to 1 mm3, representing 74% of all material discontinuities of the tested casting batch. This is a bad sign for the stability of the process, and preventive action is recommended to restore the normal distribution of the graph. 

**Figure 10:**
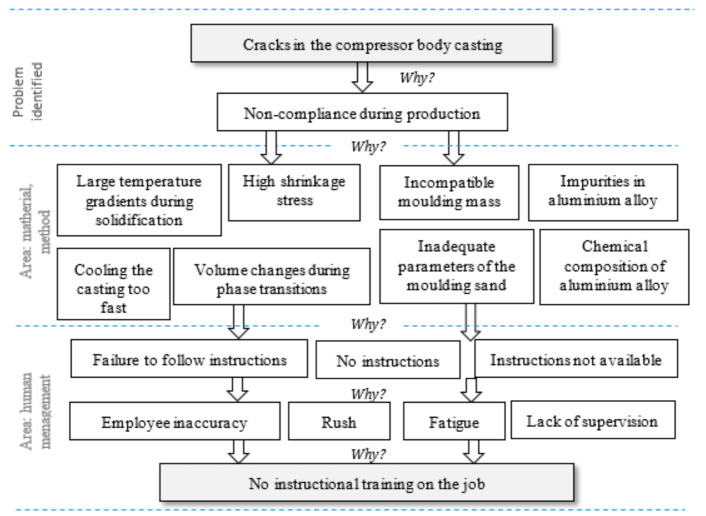
The 5WHY method for the problem of the presence of cracks in the compressor body casting.

Due to a significant number of out-of-tolerance cracks, actions were undertaken to identify the causes of the problem. The 5WHY method was used for this purpose (Figure 10).

**Figure 11:**
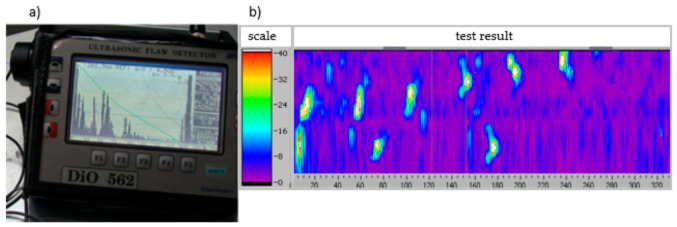
Example of the results of: (**a**) ultrasonic inspection and (**b**) eddy current inspection, indicating the presence of lumps in an aluminium casting.

Based on the analysis (Figure 11), the root cause of the cracks in the compressor body castings was the evaporator’s lack of proper qualifications due to a lack of instructional training on the job. The identified cause was classified in the human/management area.

Considering another non-conformity from group A—critical non-conformities—Figure 11 shows an example of a result from an ultrasonic and eddy current test indicating the presence of cracks.

The area examined was 210 mm thick, which can be seen as a high echo just beyond the discontinuity of the material. The material discontinuity (void) was located at a depth of 201.9 mm, with a size corresponding to a flat area of 3.8 mm. The remaining ba-dated area on the defect score was imaged with low structure noise, well below the evaluation curve.

**Figure 12:**
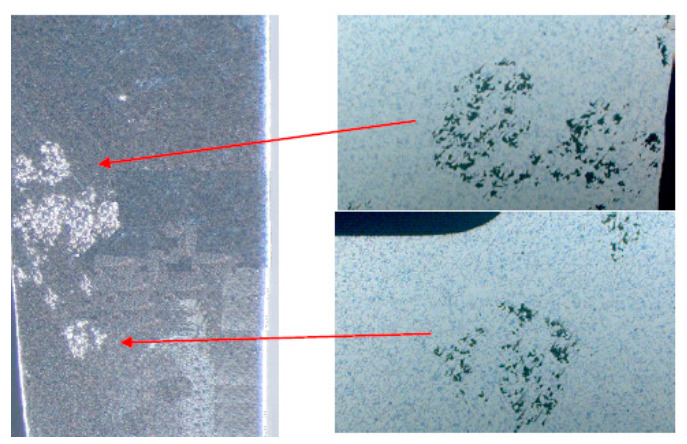
Macro and microstructure of non-conformity area in compressor body casting cracks in AlCu4Ti alloy material.

In a further step, samples were prepared for metallographic testing to better identify discrepancies and the possibility of observing discontinuities. Samples were made from defective areas of the casting on a metallographic cutter and then embedded in resin. Further steps were analogous to the previously presented sample preparation. The result of the observation in the area of rows is shown in Figure 12.

**Figure 13:**
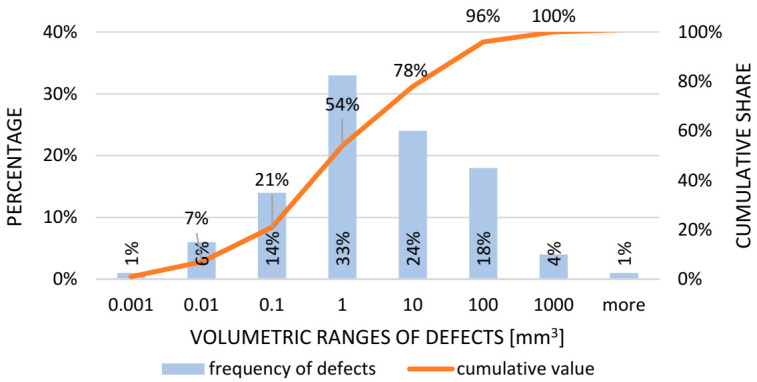
Histogram showing the frequency of occurrence of the specified groups of defects (rutting).

As part of an in-depth analysis of the size of discontinuities located inside the tested castings, a histogram was developed showing the distribution of defects as a function of frequency of occurrence and its accumulation (Figure 13).

Figure 12 shows that the most significant number of internal discontinuities (rows) generated during the compressor body casting occurs in the size of about one mm3 (33%). Together with defects in the range of 0.001 to 1 mm3, the discontinuities constituted 54% of all of the discontinuities in the material of the tested batch of castings. The data indicate a loss of process stability. In this situation, corrective action must be taken to increase the castings’ quality level and restore the normal distribution of the graph. 

Performing a histogram helped determine the type of ripples present in the castings. In the examined batch of products, lumps are most often localised. This type of rut is characterized by relatively large "voids" concentrated in specific areas of the casting. In the case of the casting under consideration - in different parts of the product, which are separated from the power source during their solidification by previously coagulated walls.

**Figure 14:**
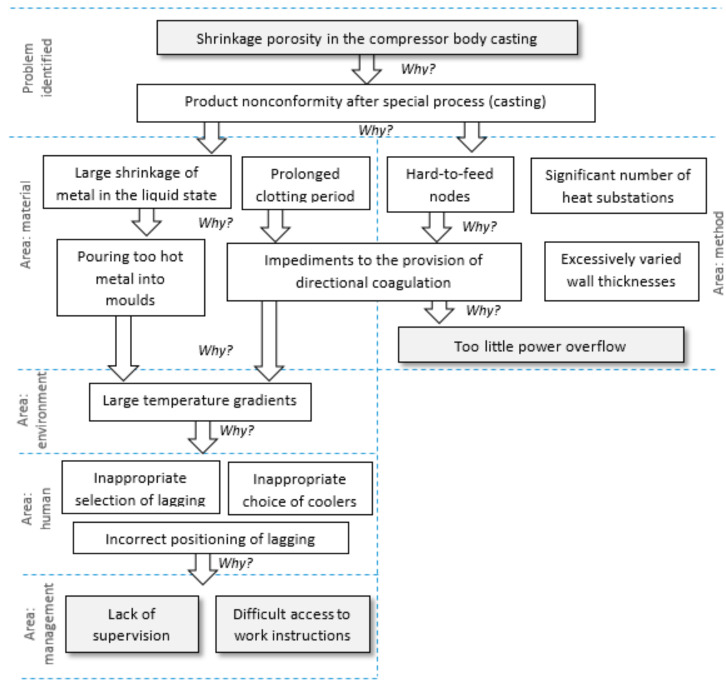
The 5WHY method for the problem of the presence of ripples in a compressor body casting.

In order to take effective action appropriate to the quality problem, it was necessary to identify the root cause of the problem. For this purpose, the 5WHY method was used, as shown in Figure 14. 

Among the answers given during the implementation of the 5WHY method, it was possible to distinguish the following areas: material, method, environment, human, and management. The qualitative problem concerning the presence of local rows proved to be complex. One of the reasons for the presence of lumps in the compressor body casting was the insufficient number of feed ingots, which significantly impeded directional solidification. The second reason was the lack of supervision and demanding access to job instructions; as a result, employees often made an inappropriate selection of laggings or coolers, and inappropriately placed the laggings. 

Reassuming the results of particular steps of the model of diagnosing and searching for discrepancies in the casting, the problem was identified as the decrease in the quality level of aluminium castings, and through the analyses, the source of the problem was found:

- the root cause of cracks in the castings of the compressor body was the lack of appropriate evaporator qualifications due to the lack of instructional training at the workplace; and
- the source cause of local government incidents was the insufficient number of feeders and the lack of supervision, and difficult access to the workplace instructions.

After completing the following steps indicated in the model of diagnosing and searching for non-conformities in the aluminium casting, it was possible to control a specific batch of products, but also to identify a group of critical non-conformities, characterize them, and indicate potential causes of a quality problem—a drop in the level of quality of aluminium castings. The proposed model of an integrally configured method allows us to go beyond passive control towards analyses leading to the identification of the root cause of the problem. 

## 5. Conclusions

The dynamically changing market, the development of enterprises, and growing customer expectations generate increased production, and the main goal is to achieve customer satisfaction [2,3,7,22]. In this context, it is essential to ensure the offered products’ appropriate quality [70,71,81]. For this reason, it is reasonable to develop a universal model for diagnosing and searching for non-conformities in aluminium castings. Based on the verification of the proposed model, it can be concluded that:

- the model allows you to go beyond the area of passive control (diagnostics and analysis);
- the model integrates selected methods of NDT detection, thus reducing the level of diagnostic uncertainty;
- the model enables quick flaw detection of aluminium castings;
- the model makes it possible to identify the root causes of a qualitative problem, thanks to which it is possible to propose practical corrective actions;
- the model enables the collection of information on quality inconsistencies specific for a given casting;
- the methods used (ultrasonic testing and eddy current testing) are complementary;
- integrating a series of diagnostic tests and quality management tools in succession increases the level of effectiveness of analyses of the existing quality problems;
- application of the proposed methodology enables control and analysis at various stages of the production process;
- the use of the model will allow for taking thoughtful improvement actions; and
- the implementation of the diagnostic and analytical steps indicated in the model is relatively cheap.

The expected benefits of implementing the proposed model include:

- manufacturing of products free of non-conformities;
- manufacturing of products in line with customer requirements;
- elimination of waste in the form of quality defects, overproduction, and waiting;
- reduction of production costs;
- conscious and quick reaction in the event of loss of stability of the manufacturing process;
- increase in customer satisfaction;
- identification of a critical group of non-conformities (in terms of frequency and effects); and
- acceleration of the decision-making process in the area of improvement activities.

Future research directions will concern the implications of the presented methodology of non-compliance analysis and quality management of manufactured products concerning other products in the analysed enterprise for a larger batch of products. Additionally, there are also plans to verify the model in other foundry companies. 

## 6. Summary

In the past, the focus was on automation to simply increase productivity and reduce costs. Today, the goal of automation has shifted to broader issues. Highly automated processes ensure high quality at a constant level if this involves a high degree of (automated) monitoring and control [122,123,124,125]. Meanwhile, the current quality control process through non-destructive inspection (NDT) is used to access the inspection procedure and service management diagnosis, followed by the NDT feedback technical assistance. Accordingly, the development of compounds-related nonionizing processes is related to the test methods. This is how an industrial production servicing service for integration, automation, control, and profitability is happening now.

The research aimed to develop a model of an integrally configured method of diagnosing and searching for non-conformities in aluminium castings, consisting of the integration of non-destructive testing and successive quality management techniques to be used in the framework of inter-operational quality control of the surface condition of castings. In the study, diagnostic tests (visual and ultrasonic tests and the method of eddy currents) were carried out in the quality control of the compressor body casting used in mining, and their analysis was carried out using an integrally configured methodology based on the following techniques: Pareto–Lorenz diagram correlated with the ABC method, histogram, and 5WHY method. The application of the presented methodology contributed to identifying the causes of non-compliance in the products and, consequently, to the elimination of non-compliant castings. Ultrasonic and eddy current defectoscopy and micro spray observation followed by Pareto–Lorenz analysis correlated with the ABC method allowed for the identification of the most severe type of non-compliance cracks in the castings. The occurrence of these discontinuities disqualifies the product. In order to define the problem of discontinuity of castings, a histogram and 5WHY analysis were performed, which allowed us to identify the root cause of non-compliance large temperature gradients during solidification caused by the inadequate management of employee qualifications. 

The applied diagnostics with the use of non-destructive testing in combination with quality management methods complement each other to a large extent and, unlike the use of single non-destructive testing or quality management instruments (which is the case in most studies on the quality control of castings), it enables a comprehensive analysis of the problem, reducing the level of diagnostic uncertainty. The proposed methodology may be a component of the methods that support quality management. Integrally correlated traditional tools can be useful when included in a chain of analyses, where the output of one tool is the entrance to another quality management method. For this reason, further research will be related to the implication of the proposed sequence of the analysis of the non-compliance of the casting within the remaining premises of the company and the products manufactured there. Each enterprise may conduct quality control differently, but the proposed sequence of methods for analysing the causes of non-compliance of products is a valuable and effective way of analysing product quality problems, which may be practised in various enterprises.

## Figures and Tables

**Table 1:** Mechanical properties of AlCu4Ti alloy [122].

Parameter	Wartość
Rm-Tensile strength (MPa)	360–400
R_p0.2_ 0.2% proof strength (MPa)	210–250
A-Min. elongation at fracture (%)	12–20
Brinell hardness (HBW)	90–120

**Table 2:** The chemical composition of AlCu4Ti alloy [122].

Pierwiastek	Si (%)	Fe (%)	Cu (%)	Mn (%)	Mg (%)	Zn (%)	Ti (%)	-
Wartość	0.15	0.15	4.2–5.2	0.01–0.5	0.03	0.07	0.15–0.25	Al-remainder

## Data Availability

Data sharing not applicable.
